# Antibiotic Resistance and Biofilm‐Forming Capacity of Oral Biofilm Bacteria in Patients With Periodontitis and Healthy Individuals

**DOI:** 10.1002/mbo3.70400

**Published:** 2026-09-08

**Authors:** Nils Werner, Marie Schöffel, Annette Wittmer, Klaus Pelz, Kirstin Vach, Cornelia Frese, Christiane von Ohle, Diana Wolff, Fabian Cieplik, Ali Al‐Ahmad

**Affiliations:** ^1^ Department of Conservative Dentistry, Periodontology and Digital Dentistry LMU University Hospital, LMU Medizin, LMU Munich Munich Germany; ^2^ Private Practice Poing Germany; ^3^ Department of Operative Dentistry and Periodontology, Center for Dental Medicine, Medical Center & Faculty of Medicine University of Freiburg Freiburg Germany; ^4^ Department of Medical Microbiology and Hygiene, Faculty of Medicine University of Freiburg Freiburg Germany; ^5^ Institute for Medical Biometry and Statistics, Faculty of Medicine Medical Center ‐ University of Freiburg, University of Freiburg Freiburg Germany; ^6^ Centre for Data Science Eberswalde University for Sustainable Development Eberswalde Germany; ^7^ Department of Conservative Dentistry, Clinic for Oral, Dental and Maxillofacial Diseases University Hospital Heidelberg Heidelberg Germany; ^8^ Department of Conservative Dentistry, Periodontology and Endodontology University Centre of Dentistry, Oral Medicine and Maxillofacial Surgery, University Hospital Tübingen Tübingen Germany

**Keywords:** antibiotic resistance, antibiotics, biofilm, periodontitis

## Abstract

This study aimed to analyse antimicrobial resistance (AMR) in bacterial isolates from subgingival biofilms of patients with periodontitis and supragingival biofilms of orally healthy individuals, and to explore the association between biofilm‐forming capacity and AMR. Three hundred and forty‐seven bacterial isolates from 44 patients were analysed. Bacterial isolates were obtained from subgingival/supragingival biofilm and identified using MALDI‐TOF mass spectrometry. Antibiotic susceptibility was evaluated by the Kirby‐Bauer test, the E‐test, and a β‐lactamase activity assay, and biofilm formation capacity was assessed using the gentian violet assay. The 44 participants (median age 28.0 [25.0; 55.0]) were stratified into untreated generalised stage III/IV periodontitis (*n* = 21) and orally healthy (*n* = 23) groups. Among 347 isolates, 74.4% formed biofilms. AMR was generally higher in isolates from orally healthy subjects (77.7% vs. 59.1% in periodontitis patients, *p* < 0.001) and females (73.2% vs. 60.6% in males, *p* = 0.026). Multivariate binary logistic mixed models linked biofilm formation to AMR (OR: 1.66 CI: [1.12, 2.49]; *p* = 0.045 for severe biofilm formers and OR: 1.93 CI: [1.31, 2.83]; *p* = 0.004 for moderate biofilm formers). Phenotypic antimicrobial resistance was thus commonly detected throughout the cohort and was at least as frequent in orally healthy participants as in patients with periodontitis. Because the orally healthy group was substantially younger, this contrast is confounded by age and smoking and cannot be interpreted as an independent effect of periodontal status. Furthermore, this study might indicate that biofilm‐forming isolates may exhibit increased antibiotic resistance.

## Graphical abstract

Key finding: Antimicrobial resistance was common across all groups and associated with biofilm‐forming capacity, an association that remains observational and species‐confounded.

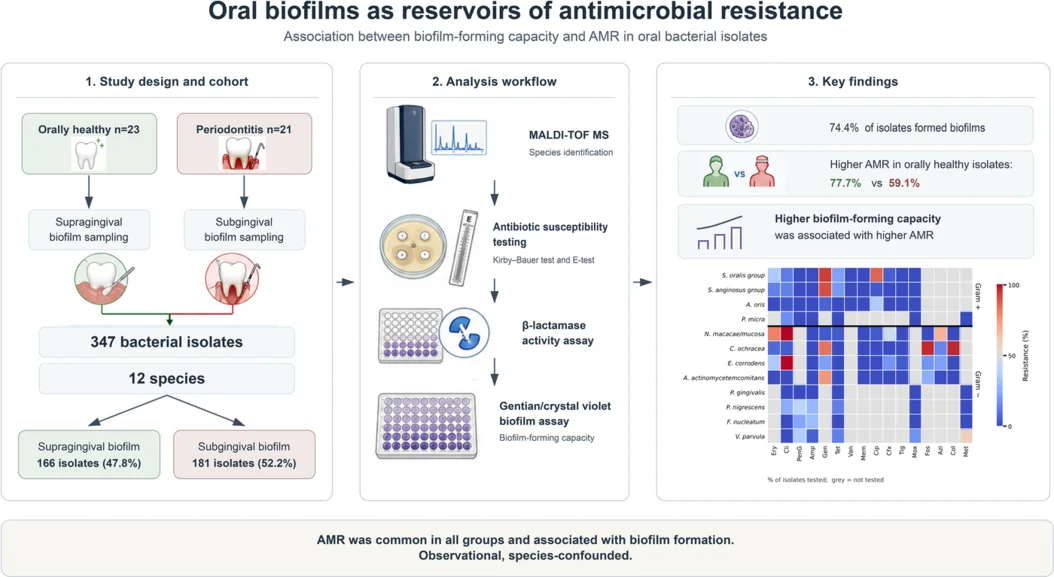

## 1 Introduction

Antimicrobial resistance (AMR) is one of the most significant global health challenges, with an estimated 10 million deaths per year projected to be associated with AMR by 2050 (O'Neill 2016). The oral cavity is considered to be a major reservoir for AMR (Al‐Ahmad et al. 2014; Anderson et al. 2023; Daller et al. 2025; Larsson and Flach 2022; Roberts and Mullany 2010), as emphasized by several statements from key stakeholders (WHO, FDI, and IADR) in 2023–2026 (Thompson et al. 2026; Thompson et al. 2025; Thompson et al. 2023). Furthermore, dentists frequently bear responsibility for the prescription of antibiotics (Thompson et al. 2025). In 2024, the number of patients who received antibiotics from dentists in Germany totalled 2,325,500, representing approximately 13.9% of the total number of antibiotic prescriptions issued that year (Cirkel et al. 2025). Periodontitis, a non‐communicable inflammatory disease, has been observed to affect 9.8% of the global population in its severe manifestation (Bernabe et al. 2020; Nascimento et al. 2024).

Periodontitis is initiated and maintained by a dysregulated immune response against a dysbiotic subgingival biofilm (Hajishengallis 2015; Hajishengallis et al. 2020). According to recent etiologic models, the developing inflammation further promotes and amplifies dysbiosis, ultimately resulting in a self‐perpetuating vicious circle (Marsh 1994; Van Dyke et al. 2020). The primary periodontal therapy can be combined with the prescriptions of various systemic antibiotics (Hagenfeld et al. 2023; Sgolastra et al. 2012; Teughels et al. 2020; van Winkelhoff et al. 1989). In this context, the current guidelines have already been developed considering antibiotic stewardship; consequently, it should be exclusively contemplated for young patients afflicted by generalised, rapidly progressing periodontitis (Sanz et al. 2020). This is especially important because of severe side effects and because the amount of AMR has increased in periodontitis‐associated bacteria over the last few decades (Jepsen and Jepsen 2016; Rams et al. 2023).

Nevertheless, the presence of AMR has been observed not only in patients with periodontitis, but also in subjects who are orally and systemically healthy (Anderson et al. 2023; Arredondo et al. 2020; Meštrović et al. 2025). In addition, it has been demonstrated that acquired resistance can be transferred horizontally through gene transfer by mobile elements such as plasmids or bacteriophages among bacteria (Ellabaan et al. 2021). This process is further enhanced by the distinct arrangement of different oral bacterial species in a biofilm (Marsh and Zaura 2017). Consequently, a correlation between biofilm formation capacity and resistance properties of bacteria appears plausible and has been observed for individual extraoral bacteria, yielding somewhat controversial results (Al‐Shamiri et al. 2021; Bahador et al. 2018; Qi et al. 2016; Ranjbar and Farahani 2019). A preceding study demonstrated that most isolates exhibiting resistance or significantly elevated MIC values from bacterial isolates from endodontically treated teeth were classified as moderate or strong biofilm producers (Al‐Ahmad et al. 2014). Furthermore, endodontic *Enterococcus faecalis* isolates showed increased biofilm forming capacity upon exposure to subinhibitory antibiotic concentrations (Bernardi et al. 2021).

This study aimed to analyse AMR in bacterial isolates obtained from subgingival biofilms of patients with periodontitis and supragingival biofilms of orally healthy individuals, and to explore whether biofilm‐forming capacity is associated with AMR. In addition, we investigated the presence of β‐lactamase enzymes as a potential mechanism contributing to resistance to β‐lactam antibiotics and characterised the resistance profiles and biofilm‐forming capacity of the oral bacterial isolates.

## 2 Materials and Methods

### 2.1 Study Design and Source of Data

This cross‐sectional pilot study was approved by the Internal Review Boards of the Universities of Freiburg, Heidelberg and Tübingen (references: 604/16; S‐652/2016; 863/2016BO2). The study was registered in the German Clinical Trials Register (DRKS00013119) and complies with the ethical principles proposed by the Declaration of Helsinki. The study description follows the guidelines for Strengthening and Reporting in Observational Studies (STROBE) (von Elm et al. 2008).

### 2.2 Study Population

**Figure 1:**
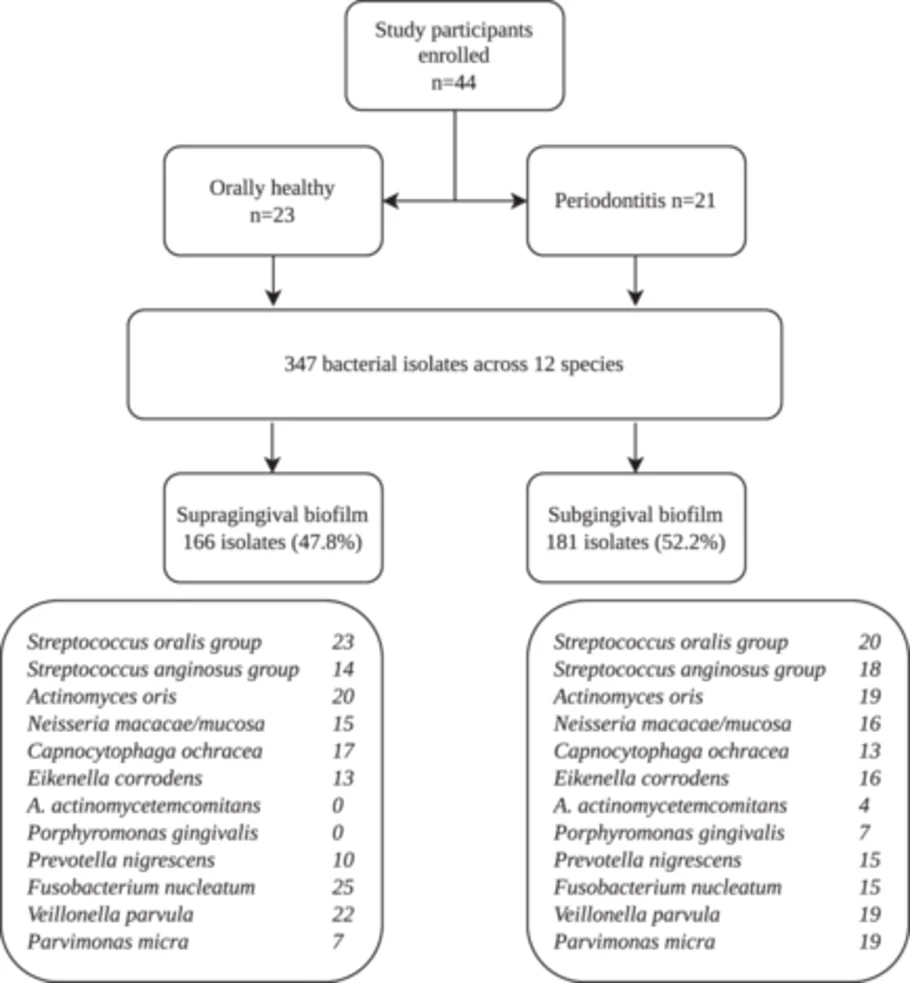
Flow diagram of the study population and bacterial isolates according to the STROBE recommendations for cross‐sectional observational studies.

This study investigated bacterial samples obtained from 44 patients recruited from the patients of all three study centers (Figure 1). All patients provided written informed consent for the study protocol. Anamnestic health information was collected, including medical history, smoking status, previous antibiotic treatment, and history of hospitalisation. Group allocation to either the orally healthy or periodontitis group was based on a comprehensive dental and periodontal examination. To be eligible for inclusion in the study, patients had to meet the following criteria: (1) age ≥ 18 years; (2) diagnosis of stage III or IV periodontitis according to the current classification (Tonetti et al. 2018) for group A, or periodontal health with a decayed‐missing‐filled index of zero for group B; (3) general health. Exclusion criteria were: (1) Pregnancy, (2) Previous periodontal treatment within 2 years before study enrolment, (3) Current participation in supportive periodontal care (SPC), (4) supragingival plaque removal during the last 6 months, (5) untreated open carious lesions, (6) antibiotic therapy during the last 6 months, (7) medications with known side effects on gingival structures, (8) pathological changes to the oral mucosa. The proportion of smokers was limited to a maximum of 20% per group to minimize disproportionate confounding by smoking on the oral microbiome and periodontal status while preserving external validity.

### 2.3 Sample Collection and Preparation

All patients were instructed not to perform interproximal cleaning for 72 h prior to sample collection and not to perform oral hygiene for 24 h prior to sample collection. This interval was required to allow sufficient supragingival plaque to accumulate for sampling in the periodontally healthy group and was applied to both groups to ensure identical pre‐sampling conditions. In subjects with periodontal disease, subgingival plaque was collected from three periodontal pockets with probing depths between 4 and 8 mm, after careful removal of supragingival plaque. Three sterile paper points (ISO 25, taper 04, ROEKO, Langenau, Germany) were sequentially inserted into each periodontal pocket and left in place for 20 s. In healthy subjects, no periodontal pockets suitable for subgingival sampling were present in the study protocol used, and supragingival plaque was therefore collected from at least one tooth surface per quadrant with a curette. All samples were pooled on the patient level and transferred into sterile cryogenic tubes (Nunc, Thermo Fisher Scientific, Waltham, MA, USA) pre‐filled with 1.5 mL of reduced transport fluid (RTF) and stored at −80°C until further processing.

After preparing the serial dilution series, the samples were plated on Columbia blood agar (CoBl) (aerobic, 5%–10% CO_2_, 37°C, 4–5 days) and yeast‐cysteine blood agar (YCB) (anaerobic, 5% CO_2_, 85% N_2_, 10% H_2_, 37°C, 10–12 days) using a GENbox atmosphere generator (BioMérieux, Marcy‐l'Étoile, France) in an anaerobic jar. Morphologically distinct colonies were subcultured (CoBl, 24 h; YCB, 48 h). Following cultivation of the monocultures, colonies were first characterized morphologically and subsequently assigned to their respective species, using matrix‐assisted laser desorption/ionization time‐of‐flight mass spectrometry (MALDI‐TOF MS). Bacterial species from diverse taxonomic families were selected in order to represent the oral bacterial flora in periodontitis patients and healthy controls.

### 2.4 Antimicrobial Susceptibility Testing

Sixteen antibiotics (ampicillin, azithromycin, cefuroxime, ciprofloxacin, clindamycin, colistin, erythromycin, fosfomycin, gentamicin, meropenem, metronidazole, moxifloxacin, penicillin G, tetracycline, tigecycline, and vancomycin) were selected for testing in vitro susceptibility. Antimicrobial susceptibility was first assessed using the disc diffusion method (Kirby–Bauer) according to EUCAST guidelines to screen for possible resistance (European Committee on Antimicrobial Susceptibility Testing 2022a). Briefly, bacterial suspensions were prepared in 0.9% NaCl to a turbidity equivalent to McFarland 0.5 (for aerobes) or adjusted visually for anaerobes. Inoculated MHBA (Müller‐Hinton‐Horseblood‐Agar) plates were prepared either by swabbing (aerobes) or by flooding BBA plates (Brucella Broth Agar) (anaerobes), followed by placement of antibiotic‐impregnated discs. Plates were incubated for 16–20 h at 37°C under aerobic or 48–72 h under anaerobic conditions. CoBl agar was used for selected fastidious species. Inhibition zone diameters were measured with calipers and interpreted according to EUCAST and further literature (Deutsches Institut für Normung e. V. 2000; European Committee on Antimicrobial Susceptibility Testing 2022a; Gales et al. 2001; Nagy et al. 2015); for *V. parvula*, for which no EUCAST zone diameter criteria are available, species‐specific criteria were derived by comparison with E‐test MIC values. For metronidazole, a zone diameter of 14 mm was applied, as opposed to 24 mm for the remaining anaerobic species; this criterion is conservative with respect to the reported finding, as application of the 24 mm criterion would classify additional isolates as resistant. Disc diffusion served as a screening step. Isolates with an inhibition zone diameter at or below a predefined species‐ and antibiotic‐specific threshold were additionally tested by E‐test (gradient diffusion). The MIC was read at the lower intersection of the inhibition ellipse with the strip and interpreted according to the current EUCAST breakpoint criteria, and this interpretation determined the final classification of these isolates. Isolates with zone diameters above the threshold were classified on the basis of the disc diffusion result. All zone diameter criteria and the corresponding thresholds for E‐test confirmation are given in Table S1. Control plates were included to confirm the purity of cultures. In accordance with the current EUCAST definition, in which the category “I” denotes susceptible, increased exposure rather than an intermediate phenotype, isolates rated as intermediate were counted as susceptible.

β‐lactamase production was assessed using the chromogenic cefinase test (Nitrocefin, Becton Dickinson, Franklin Lakes, NJ, USA). One to three bacterial colonies were applied to moistened test discs, and the appearance of a red color within 5–60 min was interpreted as positive. *Staphylococcus aureus* served as positive control.

### 2.5 Biofilm Forming Capacity

The biofilm forming capacity was measured as described previously in detail (Al‐Ahmad et al. 2014; Al‐Ahmad et al. 2008). Aerobic species were suspended in 3 mL tryptic soy broth (TSB) to obtain a homogeneous suspension and cultured in a shaking water bath for 24 h. Subsequently, 20 μL of the culture was mixed with 180 μL TSB in each well of a 96‐well plate (Greiner Bio‐One, Frickenhausen, Germany), with at least four replicate wells per strain. One column containing 200 μL TSB/well served as a blank control, and *E. faecalis* was used as a positive control. Plates were incubated for a further 24 h under aerobic conditions. Anaerobic species were prepared analogously using 3 mL Gas Chromatography Hewlett–Packard broth and cultured anaerobically for 48 h before inoculating the wells. To exclude contamination with aerobic species, aliquots of each suspension were streaked on CoBl; contaminated samples were excluded and retested. Following incubation, the culture medium was discarded, and the wells were washed three times with NaCl. Plates were air‐dried for 10 min, stained with 50 μL gentian violet for 10 min, and washed thrice with distilled water. After drying at 60°C for 10 min, the dye was resolubilized with 100 μL ethanol for 20 min on a shaker. Optical density was measured at 595 nm using a microplate reader (Tecan Infinite 200, Tecan Group Ltd., Männedorf, Switzerland). A low cut‐off value was determined by adding three standard deviations of the blank to the negative control. The high cut‐off value was defined as three times the low cut‐off value (Al‐Ahmad et al. 2014). Isolates were categorized into three groups: biofilm non‐producer (C1), moderate biofilm producer (C2), and high biofilm producer (C3).

### 2.6 Sample Size

Due to the exploratory pilot design of this study, an a priori sample size calculation was not performed. The sample size was determined by the availability of bacterial isolates obtained from microbiological analysis. This approach is consistent with recommendations for exploratory pilot studies, in which the sample size serves to provide stable estimates of variability and effect direction rather than to test hypotheses with predefined power (Lancaster et al. 2004). Effect estimates from this sample are therefore exploratory and require confirmation in larger and better‐balanced cohorts.

### 2.7 Source of Bias

Clinical measurements and sample collection were performed by internally calibrated dentists across all three study centres, which may have introduced some information bias due to variability in measurement accuracy. However, this bias was considered limited, as these data were mainly used for group classification. Laboratory analyses were conducted by MS under the supervision of AAA and colleagues using standardised procedures, thereby reducing observer and information bias. Convenience sampling for participant selection introduced a relevant risk of selection bias and limits the generalisability of the findings, which should be considered when interpreting the observed AMR patterns and associations with biofilm‐forming capacity.

### 2.8 Statistical Analysis

Isolates were categorized as either resistant (with at least one detected resistance) or susceptible. Tests rated as intermediate were counted as susceptible; this applied to 82 of 3330 tests (2.5%). Data were expressed as median with interquartile range [quartile 1; quartile 3] or mean ± standard deviation for continuous variables, and categorical variables were presented as frequencies with percentages, unless stated otherwise. Group comparisons were performed using Fisher's exact test (categorical variables) and Mann–Whitney *U* test (continuous variables). A binary logistic mixed‐effects regression model was employed to investigate (i) in a univariate model the influence of demographic covariates on antibiotic resistance and (ii) in a multivariate model the relationship between biofilm‐forming capacity and antibiotic resistance. To account for clustering of bacterial isolates within patients, patient ID was included as a random effect. Sex, smoking status, and periodontal status were included as patient‐level fixed effects, and isolate Gram stain and antibiotic spectrum class as test‐level fixed effects. Age was not included because of its collinearity with periodontal status (Table 1). In case of subsequent pairwise comparisons, the method of Scheffé was applied to adjust for multiple testing. Results are presented as odds ratios (ORs) for the outcome antibiotic resistance, relative to the stated reference category, with corresponding 95% confidence intervals (CIs). A significance level of *α* = 0.05 was applied for all tests. All analyses were performed using STATA (Version 16.1, StataCorp, College Station, Texas, USA).

**Table 1:** Baseline characteristics.

Variable	Total (*n* = 44)	Periodontitis (*n* = 21)	Orally Healthy (*n* = 23)	*p* value
Age, y	28.0 [25.0; 55.0]	56.5 [49.0; 61.0]	26.0 [21.0; 27.0]	< 0.001
Sex				
Female, *n* (%)	25 (100)	9 (36.0)	16 (64.0)	0.069
Male, *n* (%)	19 (100)	12 (63.2)	7 (36.8)	
Smoking Status				
Non‐smokers, *n* (%)	33 (100)	11 (33.3)	22 (66.7)	< 0.001
Smokers, *n* (%)	11 (100)	10 (90.9)	1 (9.1)	

*Note:* Data are presented as median [q1; q3] or as *n* (%). Comparisons are performed using Fisher's exact test (categorical variables) and Mann–Whitney *U* test (continuous variables). Bold indicates statistically significant values (*p* < 0.05).

## 3 Results

### 3.1 Patient and Isolate Characteristics

The final analysis included 347 bacterial isolates obtained from 44 patients collected between September 2017 and December 2020. The median age of these patients was 28.0 [25.0; 55.0] years. The male‐to‐female ratio was 43.2/56.8%, and the smoker‐to‐non‐smoker ratio was 25.0/75.0%. Patients could be stratified into periodontitis (*n* = 21) or orally healthy (*n* = 23). The two groups differed significantly in terms of smoking status (*p* < 0.001). Additionally, the orally healthy group was significantly younger than the periodontitis group (26.0 [21.0; 27.0] vs. 56.5 [49.0; 61.0], *p* < 0.001) (Table 1). Bacterial isolates from 12 bacterial species were investigated (Figure 1). One hundred and sixty‐six (47.8%) could be extracted from the orally healthy, and 181 (52.2%) from the periodontitis patient cohort. Regarding biofilm formation, 89 (25.6%) of isolates showed little to no biofilm formation, 131 (37.8%) showed moderate biofilm formation, and 127 (36.6%) showed severe biofilm formation (Table 2). Stratified by group, 52 of 89 non‐biofilm‐forming isolates (58.4%) were obtained from orally healthy individuals, while moderate (73/131, 55.7%) and severe (71/127, 55.9%) biofilm formers were more frequently obtained from periodontitis patients.

**Table 2:** Isolate characteristics.

Variables		Sex		Smoking status		Oral status		Biofilm forming			Resistance		Cefinase	
	*n*	Male, *n* (%)	Female, *n* (%)	Non‐smokers, *n* (%)	Smokers, *n* (%)	Orally Healthy, *n* (%)	Periodontitis, *n* (%)	C1, *n* (%)	C2, *n* (%)	C3, *n* (%)	Susceptible, *n* (%)	Resistant, *n* (%)	Negative, *n* (%)	Positive, *n* (%)
All	347	142 (40.9)	205 (59.1)	256 (73.8)	91 (26.2)	166 (47.8)	181 (52.2)	89 (25.6)	131 (37.8)	127 (36.6)	111 (32.0)	236 (68.0)	331 (95.4)	16 (4.6)
*Streptococcus oralis groups*	43	22 (51.2)	21 (48.8)	34 (79.1)	9 (20.9)	23 (53.5)	20 (46.5)	0 (0.0)	19 (44.2)	24 (55.8)	1 (2.3)	42 (97.7)	43 (100.0)	0 (0.0)
*Streptococcus anginosus group*	32	14 (43.8)	18 (56.3)	22 (68.8)	10 (31.3)	14 (43.8)	18 (56.3)	2 (6.3)	21 (65.6)	9 (28.1)	3 (9.4)	29 (90.6)	32 (100.0)	0 (0.0)
*Actinomyces oris*	39	17 (43.6)	22 (56.4)	27 (69.2)	12 (30.8)	20 (51.3)	19 (48.7)	0 (0.0)	1 (2.6)	38 (97.4)	25 (64.1)	14 (35.9)	39 (100.0)	0 (0.0)
*Neisseria macacae/mucosa*	31	9 (29.0)	22 (71.0)	21 (67.7)	10 (32.3)	15 (48.4)	16 (51.6)	1 (3.2)	6 (19.4)	24 (77.4)	0 (0.0)	31 (100.0)	31 (100.0)	0 (0.0)
*Capnocytophaga ochracea*	30	9 (30.0)	21 (70.0)	24 (80.0)	6 (20.0)	17 (56.7)	13 (43.3)	1 (3.3)	24 (80.0)	5 (16.7)	0 (0.0)	30 (100.0)	30 (100.0)	0 (0.0)
*Eikenella corrodens*	29	8 (27.6)	21 (72.4)	23 (79.3)	6 (20.7)	13 (44.8)	16 (55.2)	5 (17.2)	14 (48.3)	10 (34.5)	0 (0.0)	29 (100.0)	29 (100.0)	0 (0.0)
*Aggregatibacter actinomycetemcomitans*	4	1 (25.0)	3 (75.0)	3 (75.0)	1 (25.0)	0 (0.0)	4 (100.0)	0 (0.0)	2 (50.0)	2 (50.0)	1 (25.0)	3 (75.0)	4 (100.0)	0 (0.0)
*Porphyromonas gingivalis*	7	5 (71.4)	2 (28.6)	4 (57.1)	3 (42.9)	0 (0.0)	7 (100.0)	7 (100.0)	0 (0.0)	0 (0.0)	7 (100.0)	0 (0.0)	7 (100.0)	0 (0.0)
*Prevotella nigrescens*	25	11 (44.0)	14 (56.0)	15 (60.0)	10 (40.0)	10 (40.0)	15 (60.0)	11 (44.0)	14 (56.0)	0 (0.0)	13 (52.0)	12 (48.0)	16 (64.0)	9 (36.0)
*Fusobacterium nucleatum*	40	16 (40.0)	24 (60.0)	33 (82.5)	7 (17.5)	25 (62.5)	15 (37.5)	29 (72.5)	11 (27.5)	0 (0.0)	28 (70.0)	12 (30.0)	33 (82.5)	7 (17.5)
*Veillonella parvula*	41	18 (43.9)	23 (56.1)	33 (80.5)	8 (19.5)	22 (53.7)	19 (46.3)	21 (51.2)	7 (17.1)	13 (31.7)	11 (26.8)	30 (73.2)	41 (100.0)	0 (0.0)
*Parvimonas micra*	26	12 (46.2)	14 (53.8)	17 (65.4)	9 (34.6)	7 (26.9)	19 (73.1)	12 (46.2)	12 (46.2)	2 (7.7)	22 (84.6)	4 (15.4)	26 (100.0)	0 (0.0)

### 3.2 Antibiotic Resistance

**Figure 2:**
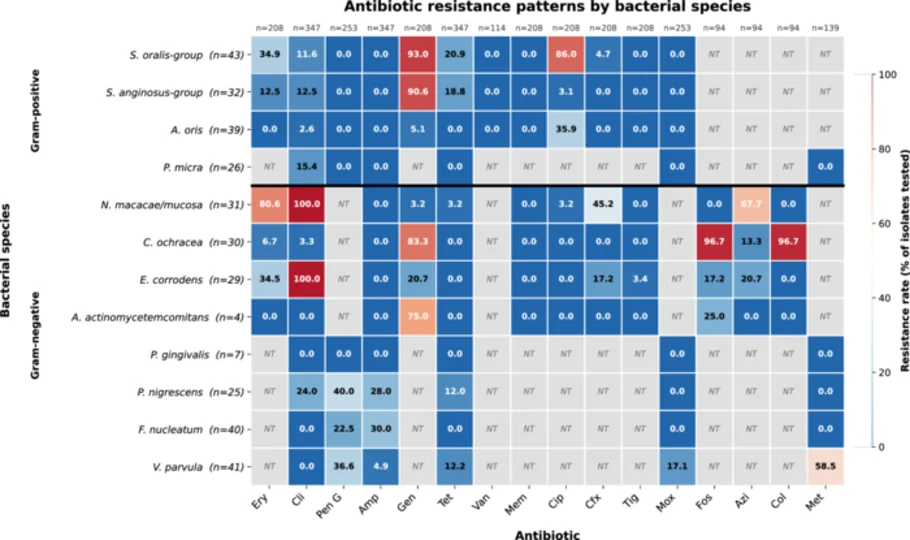
Antibiotic resistance patterns by bacterial species. Each cell shows the proportion of isolates of a given species categorised as resistant to the respective antibiotic, expressed as a percentage of the isolates of that species actually tested with that agent. Denominators are given for each species (row labels) and for each antibiotic (top axis). NT = not tested. Species are grouped by Gram stain, separated by the horizontal rule. The *S. oralis*‐group comprises *S. oralis*, *S. mitis*, *S. infantis* and *S. gordonii*; the *S. anginosus*‐group comprises *S. anginosus*, *S. intermedius* and *S. constellatus*. Antibiotics: Amp, ampicillin; Azi, azithromycin; Cfx, cefuroxime; Cip, ciprofloxacin; Cli, clindamycin; Col, colistin; Ery, erythromycin; Fos, fosfomycin; Gen, gentamicin; Mem, meropenem; Met, metronidazole; Mox, moxifloxacin; Pen G, penicillin G; Tet, tetracycline; Tig, tigecycline; Van, vancomycin. Isolates categorised as I (susceptible, increased exposure) were counted as susceptible.

Of the 347 isolates examined, 236 (68.0%) showed resistance to at least one antibiotic. Resistance to gentamicin was most common (51.0%), while resistance to the antibiotics meropenem and vancomycin was not observed. None of the periodontitis‐associated species (orange and red complexes, (Socransky et al. 1998) showed resistance to metronidazole. The only metronidazole resistance observed occurred in *V. parvula* (24 of 41 isolates, 58.5%), a member of the purple complex, which was recovered from both cohorts. Notably, 12 (30.0%) isolates from *F. nucleatum* were resistant to ampicillin (Figure 2). Further, 62 (17.9%) isolates showed resistance to more than two antibiotic classes and were therefore classified as multidrug‐resistant.

A considerable disparity in patient characteristics was identified when the number of isolates with at least one positive resistance test was considered. A higher proportion of isolates exhibiting resistance was documented in non‐smokers compared to smokers (187 (73.0%) in non‐smokers vs. 49 (53.8%) in smokers, OR: 0.43, *p* = 0.001) (Table 3). Isolates from female subjects (150, 73.2%) or orally healthy subjects (129, 77.7%) tended to demonstrate higher levels of resistance than isolates from male subjects (86, 60.6%; OR: 1.81, *p* = 0.026) or periodontitis patients (107, 59.1%; OR: 0.41, *p* < 0.001) (Table 3). Furthermore, isolates exhibiting elevated biofilm‐forming capacity possessed a substantially higher number of resistances in comparison to those that do not form biofilms (Tables 3 and 4). Notably, the univariate association between smoking and resistance (OR 0.43, *p* = 0.001) did not persist in the adjusted model (OR 1.02, *p* = 0.842), consistent with the near‐complete overlap between smoking status and periodontal status in this sample. Regarding the presence of β‐lactamases, seven *F. nucleatum* isolates and nine *P. nigrescens* isolates were identified.

**Table 3:** Resistance characteristics of bacterial isolates (univariate analysis).

Variable	Total (*n* = 347)	Susceptible	Resistant	Odds ratio (95% CI)	*p* value
Sex					**0.026**
Male	142	56 (39.4%)	86 (60.6%)		
Female	205	55 (26.8%)	150 (73.2%)	1.81 [1.07, 3.07]	
Smoking Status					**0.001**
Non‐smokers	256	69 (27.0%)	187 (73.0%)		
Smokers	91	42 (46.2%)	49 (53.8%)	0.43 [0.26, 0.72]	
Oral Status					**< 0.001**
Orally Healthy	166	37 (22.3%)	129 (77.7%)		
Periodontitis	181	74 (40.9%)	107 (59.1%)	0.41 [0.25, 0.67]	
Biofilm‐forming					
Non (reference)	89	49 (55.1%)	40 (44.9%)		
Moderate	131	32 (24.4%)	99 (75.6%)	4.79	**< 0.001**
Severe	127	30 (23.6%)	97 (76.4%)	5.25	**< 0.001**

*Note:* Data are presented as *n* (%) and Odds ratio with (95%‐confidence interval). Comparisons are performed using mixed‐effects logistic regression models. Post hoc pairwise comparisons between categories were adjusted for multiple testing using the Scheffé method. Confidence intervals are model‐based intervals for the estimated coefficients and are not available for the pairwise contrasts between non‐reference categories, which are therefore reported as odds ratios with adjusted *p* values only. Bold indicates statistically significant values (*p* < 0.05).

**Table 4:** Mixed‐effects binary logistic regression model (dependent variable: antibiotic resistance).

	Odds ratio	95% CI	*p* value
Biofilm forming			
Non (reference) vs. moderate	1.93	1.31, 2.83	**0.004**
Non (reference) vs. severe	1.66	1.12, 2.49	**0.045**
Moderate (reference) vs. Severe	0.86		0.408
Male (reference) vs. female	1.15	0.96, 1.38	0.123
Non‐smoking (reference) vs. smoking	1.02	0.83, 1.26	0.842
Orally Healthy (reference) vs. periodontitis	0.59	0.48, 0.73	**< 0.001**
Isolate Gram stain negative (reference) vs. Isolate Gram stain gram positive	0.47	0.38, 0.58	**< 0.001**
Antibiotic spectrum class			
Gram‐positive (reference) vs. Gram‐negative	1.67	1.22, 2.29	**0.017**
Gram‐positive (reference) vs. Broad spectrum	0.19	0.14, 0.24	**< 0.001**
Gram‐positive (reference) vs. Anaerobe	1.16	0.90, 1.50	0.721
Gram‐negative (reference) vs. Broad spectrum	0.11		**< 0.001**
Gram‐negative (reference) vs. Anaerobe	0.70		0.189
Broad spectrum (reference) vs. Anaerobe	6.25		**< 0.001**

*Note:* Data are presented as Odds ratio with 95%‐confidence interval (CI). Comparisons are performed using mixed‐effects logistic regression adjusted for clustering by patient (*n* = 44 clusters). Post hoc pairwise comparisons between categories were adjusted for multiple testing using the Scheffé method. Confidence intervals are model‐based intervals for the estimated coefficients and are not available for the pairwise contrasts between non‐reference categories, which are therefore reported as odds ratios with adjusted *p* values only. Bold indicates statistically significant values (*p* < 0.05).

Mixed‐effects modelling confirmed the increased burden of AMR in biofilm‐forming isolates (OR: 1.66, 95% CI: 1.12, 2.49, *p* = 0.045 for severe biofilm formers, and OR: 1.93, 95% CI: 1.31, 2.83, *p* = 0.004 for moderate biofilm formers). The analysis accounts for potential confounders related to patient characteristics (sex, smoking, and periodontitis) as well as test‐specific variables (antibiotic spectrum and Gram staining). Significant confounding effects were observed for periodontitis (OR: 0.59, 95% CI: 0.48, 0.73, *p* < 0.001). Additionally, the type of antibiotic tested showed significant differences in AMR (Table 4). Post hoc pairwise comparisons revealed that broad‐spectrum antibiotics had substantially lower resistance rates compared to both Gram‐positive‐effective (OR = 0.19, *p* < 0.001) and Gram‐negative‐effective antibiotics (OR = 0.11, *p* < 0.001), while anaerobe‐effective antibiotics showed significantly higher resistance compared to broad‐spectrum antibiotics (OR = 6.25, *p* < 0.001).

## 4 Discussion

The objective of the present study was to examine bacterial isolates from periodontitis patients and orally healthy individuals with regard to antibiotic resistance and to assess whether biofilm‐forming capacity is associated with resistance profiles. The findings support the notion that both the supragingival biofilm of orally healthy individuals and the subgingival biofilm may serve as reservoirs of AMR. Furthermore, the results indicate a positive association between higher biofilm‐forming capacity and antibiotic resistance. However, several potential factors, although not periodontitis itself, may also influence AMR.

Species‐level identification was performed using MALDI‐TOF MS analysis (Alizadeh et al. 2021; Anderson et al. 2014), an established, cost‐efficient alternative to PCR‐based methods (Bartsch et al. 2025; Pinto et al. 2017). Most species were detected in both groups, whereas *A. actinomycetemcomitans* and *P. gingivalis* were found only in patients with periodontitis (Hajishengallis et al. 2012; Werner et al. 2024). Phenotypic resistance was assessed using the Kirby–Bauer test and the E‐test, both of which are widely used methods that yield largely comparable results (Carvalho et al. 2022; European Committee on Antimicrobial Susceptibility Testing 2022b). To confirm resistance behaviour, the E‐test, an accepted method in clinical microbiology, was performed on all isolates that did not show clear susceptibility in the disc diffusion screening, and the resulting MIC determined their final classification. Biofilm‐forming capacity was quantified using a crystal violet assay, which measures total biomass but does not distinguish between viable cells, non‐viable cells, and extracellular matrix components. Therefore, species‐dependent differences in matrix composition may have led to overestimation of biofilm formation (Jakubovics et al. 2021). To reduce this effect, species‐specific cut‐off values were applied, as previously used to assess the association between biofilm‐forming capacity and antibiotic resistance in oral bacteria from endodontic infections (Al‐Ahmad et al. 2014).

Resistance to 16 antibiotics was analysed. Antibiotic selection was based on relevance to dental and general medicine, activity against Gram‐positive and Gram‐negative bacteria, inclusion of both established and newer agents, and documented resistance mechanisms. Compared with previous studies, this investigation assessed a broader range of bacterial species and antibiotics (Ardila et al. 2010; Jepsen et al. 2021; Rams et al. 2020, 2023). In line with these previously published articles, our analysis revealed resistance to multiple commonly prescribed antibiotics, including multiple antibiotics categorised as significant by the WHO (World Health Organization 2022). A relatively high proportion of isolates demonstrated resistance to gentamicin, particularly streptococci. Since aminoglycosides are primarily active against Gram‐negative bacilli and staphylococci, this finding is not surprising, and a combination with penicillin is recommended (Craig 2011). Notably, no resistance was found to the third‐line antibiotics vancomycin and meropenem. In total, 17.9% of the isolates exhibited multidrug resistance, defined here as resistance to more than two of the antibiotic classes tested. As the panel deliberately included agents against which several of the tested genera are intrinsically non‐susceptible, such as gentamicin in streptococci, this figure comprises both intrinsic and acquired resistance and is not directly comparable with the WHO or CDC definitions. Nevertheless, it supports the view that the oral cavity may serve as a reservoir for antibiotic resistance and underlines the need for prudent antibiotic prescribing in dentistry. Several molecular mechanisms may contribute to the prevalence of antimicrobial and multidrug resistance observed in the present cohort, including multidrug efflux pumps, altered antimicrobial penetration and tolerance mediated by the extracellular polymeric substance matrix, and quorum‐sensing‐dependent regulation of resistance gene expression and biofilm maturation. In addition, chronic exposure of oral biofilm bacteria to subinhibitory concentrations of antimicrobial agents from oral hygiene products and food preservatives may exert continuous selective pressure, thereby contributing to the emergence and maintenance of resistance in oral biofilm communities (Cieplik et al. 2019; Cieplik et al. 2025; Govindarajan et al. 2022).

Moreover, the results of our study demonstrate a higher proportion of resistant isolates in female subjects. However, there are no comparable preliminary studies on oral bacteria. A recent study analysing the gut resistome found 9% more antibiotic resistance genes in females (Salehi et al. 2025). Additionally, women are prescribed 27% more antibiotics than men during their lifetime (World Health Organization 2024). Frequent or inappropriate antibiotic use may contribute to increased resistance (Llor and Bjerrum 2014), and could partly explain the higher AMR levels observed in female subjects. In addition, sex‐related differences in the oral microbiome may be relevant. Biofilms exposed to sex steroid hormones, including progesterone, have shown increased proteolytic activity (Cornejo Ulloa et al. 2023), and large‐scale metagenome‐wide association data suggest interactions between sex hormones and oral microbiome composition (Liu et al. 2023). Further, our results demonstrated a higher proportion of resistance in isolates from non‐smokers. This somewhat contradicts the literature, although studies of oral bacteria are missing in this area as well. However, there is a higher prescription of antibiotics in smoking patients (Steinberg et al. 2016), which could lead to AMR (Llor and Bjerrum 2014), and studies have also shown that cigarette smoking might lead to an increased burden of AMR (Witteman et al. 1993; Xu et al. 2020). Nevertheless, smoking is known to shape the oral microbiome and is associated with a less diverse, distinct bacterial community even in periodontally healthy or only mildly diseased individuals (Al Kawas et al. 2021). Only one of the 23 periodontally healthy participants was a smoker, so that this group provides essentially no information from which a smoking effect could be estimated. Smoking status was therefore retained as a covariate but is not interpreted as an independent finding. Although lower AMR levels were observed in isolates from patients with periodontitis than from orally healthy individuals, the two groups differed substantially in age (median 56.5 vs. 26 years) and in smoking status (10 of 21 vs. 1 of 23 smokers), and residual confounding of this contrast cannot be excluded. Due to collinearity, age was not included in the multivariable model. The higher AMR levels observed in younger, orally healthy individuals may reflect age‐ or cohort‐related differences in antibiotic exposure, intrafamilial transmission of resistant bacteria, or other unmeasured factors (Carvalho et al. 2022). However, a metagenomic study evaluating the oral resistome showed similar results, concluding that the lower level of AMR in periodontitis might additionally be explained by the lower diversity and distinct microbial composition in periodontitis (Anderson et al. 2023; Gager et al. 2023). Nevertheless, the comparability of genotypic resistance to phenotypic resistance is limited; it is frequently noticed that not all antibiotic‐resistant genes confer antibiotic resistance (Anderson et al. 2023; Auer et al. 2022; Crofts et al. 2017). Furthermore, gene expression in biofilm‐associated bacteria can differ substantially from planktonic cultures, and certain resistance mechanisms may be biofilm‐induced or remain silent in vitro, which should be considered when interpreting the present phenotypic resistance findings (Ciofu et al. 2022; Hall and Mah 2017). Nevertheless, several studies have reported a high prevalence of genotypic resistance patterns across the resistome of healthy individuals. Particularly striking are the findings by Carr et al. who reported a higher abundance of AMR genes in the oral cavity than in the gut (Carr et al. 2020). Further whole‐genome sequencing and resistome analyses of healthy adults have demonstrated a substantial prevalence of resistance determinants against macrolides, lincosamides, streptogramins, and tetracyclines (Caselli et al. 2020). The stable supragingival biofilm of orally healthy individuals may provide ecological conditions that support the persistence of biofilm‐forming and resistant phenotypes and may facilitate the exchange of mobile resistance determinants, even in the absence of acute selective pressure (Sukumar et al. 2016). Additionally, a high prevalence of β‐lactamases was detected in the periodontitis‐associated bacterium *F. nucleatum*; as amoxicillin features in the standard combination regimens, their capacity to hydrolyse the β‐lactam locally is of direct clinical relevance (Werner et al. 2025).

To the best of our knowledge, the capacity of subgingival and supragingival bacteria to form biofilms and their association with phenotypic antibiotic resistance have not been sufficiently studied to date. For this reason, we utilized a static microtiter plate model to evaluate biofilm‐forming capacities (Al‐Ahmad et al. 2014; Al‐Ahmad et al. 2008). Although this method does not reflect in vivo conditions, it met the requirements of the present study, which focused on the representation of biofilm formation by the isolates. We found an increased prevalence of phenotypic resistance in biofilm formers. This association has already been demonstrated by a study conducted by our working group on bacterial isolates from root canals (Al‐Ahmad et al. 2014). In our findings, this result was even robust against adjusting for confounding variables.

It is essential to acknowledge that the present study is subject to several limitations. Notably, the local determination of this observational study affects the generalizability of the findings. The overall sample size at the patient level is relatively small; nevertheless, a high and diverse number of bacterial isolates could be obtained from the subjects, which is why the main results are focused on the isolate level, where meaningful within‐species comparisons remain largely feasible. Sampling site and collection instrument differed between the two groups, and supragingival and subgingival plaque represent distinct ecological niches that are not directly comparable. Restricting comparisons to isolates of the same species reduces, but does not eliminate, this influence, since the probability of recovering particular strains or resistant subpopulations may itself differ between the two niches, and two species (*A. actinomycetemcomitans* and *P. gingivalis*) were recovered from the periodontitis group only. Standardisation of the in vitro biofilm assay likewise does not remove selection introduced during sampling and isolation. Different sampling sites are therefore a potentially important source of selection bias in this study. The Gram stain of the isolate was included in the model, which accounts for part of the compositional difference between the two niches, but residual selection bias cannot be excluded (Arredondo et al. 2025; Arredondo et al. 2020). It is important to acknowledge the substantial age and smoking difference between the study groups and significant patient‐level differences between the stratified groups. Although covariate adjustments were made for smoking and sex, residual confounding cannot be excluded. In particular, the younger age of the orally healthy group limits extrapolation of these findings to age‐matched healthy adults. The standardised interval without oral hygiene before sampling allowed a short period of undisturbed plaque accumulation, which may have introduced variability in the composition of the sampled biofilm, particularly in the periodontally healthy group, where supragingival plaque was sampled. Species identity could not be modelled directly, as four of the twelve species showed no within‐species variation in resistance. Species was therefore represented by Gram stain, which is a coarse proxy, and residual confounding by species remains. Because biofilm‐forming capacity is itself strongly species‐dependent, the observed association between biofilm category and resistance may partly reflect species identity rather than a within‐species relationship. Furthermore, molecular mechanisms underlying antibiotic resistance, such as efflux pump activity, extracellular polymeric matrix composition, and quorum sensing, were not investigated and should be addressed in future mechanistic studies.

## 5 Conclusions

The rationale for the use of systemic antibiotics in periodontal therapy has been a matter of debate for many years. Considering AMR, a targeted use of antibiotics in dentistry is recommended. However, AMR may not be relevant only to patients with periodontal disease but also to orally healthy and younger individuals, among whom a notable burden of antibiotic resistance was already observed in the present cohort. As the two groups differed substantially in age, this contrast is confounded by age and smoking and cannot be interpreted as an independent effect of periodontal status. Within the limitations of this study, biofilm‐forming capacity was associated with antibiotic resistance among the bacterial isolates examined. This association is observational and is confounded by species identity, and it should not be interpreted as causal.

## Author Contributions

**Nils Werner:** writing – original draft, visualization. **Marie Schöffel:** investigation, methodology, writing – review and editing. **Annette Wittmer:** methodology, investigation, writing – review and editing. **Klaus Pelz:** methodology, investigation, writing – review and editing. **Kirstin Vach:** formal analysis, data curation, writing – review and editing. **Cornelia Frese:** conceptualization, writing – review and editing. **Christiane von Ohle:** conceptualization, writing – review and editing, supervision. **Diana Wolff:** conceptualization, writing – review and editing, supervision. **Fabian Cieplik:** writing – review and editing, resources. **Ali Al‐Ahmad:** conceptualization, writing – original draft, supervision.

## Ethics Statement

This pilot study was approved by the ethics committees of all three participating medical faculties: Albert‐Ludwigs‐Universität Freiburg (No. 604/16), Ruprecht‐Karls‐Universität Heidelberg (No. S‐652/2016), and Eberhard‐Karls‐Universität Tübingen (No. 863/2016BO2), covering the Department of Operative Dentistry and Periodontology at Freiburg University Hospital, the Department of Conservative Dentistry at Heidelberg University Hospital, and the Department of Conservative Dentistry, Periodontology and Endodontology at Tübingen University Hospital, respectively. Informed consent was obtained from all subjects included in the study.

## Conflicts of Interest

The authors declare no conflicts of interest.

## AI Statement

Claude (Anthropic) was used for language editing and grammar checking. All scientific content, data analysis, and interpretation were performed independently by the authors.

## Supporting information


Supporting File

## Data Availability

The data that support the findings of this study are available on request from the corresponding author. The data are not publicly available due to privacy or ethical restrictions.
