# Supplementary material for: Antibiotic Resistance and Biofilm‐Forming Capacity of Oral Biofilm Bacteria in Patients With Periodontitis and Healthy Individuals
Source: Microbiologyopen. 2026 Sep 8;15(5):e70400. doi: 10.1002/mbo3.70400 (PMC13551596; doi:10.1002/mbo3.70400)
Supplement: Supplementary file 1 [file MBO3-15-e70400-s001.docx]

**Supplementary Table S1.** Disc diffusion criteria and zone diameter thresholds triggering E-test confirmation, by antibiotic and bacterial species.

| **Antibiotic** | **Disc diffusion criterion (mm)** | | **Zone diameter at or below which E-test confirmation was performed (mm)** | | | | | | | | |
| --- | --- | --- | --- | --- | --- | --- | --- | --- | --- | --- | --- |
|  | **Susceptible zone >** | **Resistant zone <** | ***S. oralis, S. anginosus group, A. oris*** | ***N. macacae/ mucosa*** | ***C. ochracea*** | ***E. corrodens*** | ***A. actinomycetem-comitans*** | ***P. gingivalis, P. nigrescens*** | ***F. nucleatum*** | ***V. parvula*** | ***P. micra*** |
| Ampicillin | 21 | 15 | 22 | 17 | 25 | 18 | 25 | 25 | 25 | 14 | 22 |
| Azithromycin | 21 | 18 | *–* | 19 | 21 | 21 | 21 | *–* | *–* | *–* | *–* |
| Cefuroxime | 26 | 26 | 25 | 20 | 25 | 25 | 25 | *–* | *–* | *–* | *–* |
| Ciprofloxacin | 23ᵇ | 19ᵇ | 21 | 25 | 25 | 25 | 25 | *–* | *–* | *–* | *–* |
| Clindamycin | 19ᶜ | 19ᶜ | 21 | 17 | 25 | 14 | 25 | 25 | 25 | 18 | 21 |
| Colistin | 14 | 12 | *–* | 15 | 16 | 18 | 18 | *–* | *–* | *–* | *–* |
| Erythromycin | 21 | 18 | 21 | 18 | 21 | 21 | 21 | *–* | *–* | *–* | *–* |
| Fosfomycin | 24 | 24 | *–* | 24 | 25 | 25 | 25 | *–* | *–* | *–* | *–* |
| Gentamicin | 17 | 17 | 17 | 20 | 17 | 17 | 17 | *–* | *–* | *–* | *–* |
| Meropenem | 22 | 14 | 22 | 25 | 25 | 25 | 25 | *–* | *–* | *–* | *–* |
| Metronidazole | 24ᵃ | 24ᵃ | *–* | *–* | *–* | *–* | *–* | 22 | 25 | 13 | 25 |
| Moxifloxacin | 19 | 19 | 19 | *–* | *–* | *–* | *–* | 22 | 22 | 17 | 22 |
| Penicillin G | 18 | 12 | 24 | *–* | *–* | *–* | *–* | 24 | 24 | 20 | 24 |
| Tetracycline | 23ᵈ | 20ᵈ | 20 | 23 | 25 | 25 | 20 | 22 | 22 | 16 | 22 |
| Tigecycline | 19 | 19 | 19 | 20 | 25 | 20 | 25 | *–* | *–* | *–* | *–* |
| Vancomycin | 15 | 15 | 14 | *–* | *–* | *–* | *–* | *–* | *–* | *–* | *–* |

All zone diameters refer to disc diffusion testing. Isolates with a zone diameter greater than the susceptible criterion were categorised as susceptible, and those with a zone diameter smaller than the resistant criterion as resistant; isolates between the two were categorised as intermediate, and where the two criteria are identical no intermediate category applies. Isolates with a zone diameter at or below the threshold given in the right-hand columns were additionally tested by E-test.

**ᵃ** For *V. parvula*, the metronidazole criteria were 14 mm (susceptible, zone >) and 14 mm (resistant, zone <)

**ᵇ** For *N. macacae/mucosa, C. ochracea, E. corrodens* and *A. actinomycetemcomitans*, the ciprofloxacin criteria were 25 mm (susceptible) and 22 mm (resistant).

**ᶜ** For *N. macacae/mucosa, C. ochracea, E. corrodens* and *A. actinomycetemcomitans*, the clindamycin criteria were 17 mm (susceptible) and 17 mm (resistant).

**ᵈ** For *P. gingivalis, P. nigrescens, F. nucleatum, V. parvula* and *P. micra*, the tetracycline criteria were 22 mm (susceptible) and 17 mm (resistant).
